# Deep Learning Based SWIR Object Detection in Long-Range Surveillance Systems: An Automated Cross-Spectral Approach

**DOI:** 10.3390/s22072562

**Published:** 2022-03-27

**Authors:** Miloš S. Pavlović, Petar D. Milanović, Miloš S. Stanković, Dragana B. Perić, Ilija V. Popadić, Miroslav V. Perić

**Affiliations:** 1 School of Electrical Engineering, University of Belgrade, Bul. Kralja Aleksandara 73, 11120 Belgrade, Serbia; petar.milanovic@vlatacom.com; 2 Vlatacom Institute of High Technologies, Milutina Milankovica 5, 11070 Belgrade, Serbia; milos.stankovic@vlatacom.com (M.S.S.); dragana.peric@vlatacom.com (D.B.P.); ilija.popadic@vlatacom.com (I.V.P.); miroslav.peric@vlatacom.com (M.V.P.); 3 Faculty of Technical Sciences, Singidunum University, Danijelova 32, 11000 Belgrade, Serbia

**Keywords:** SWIR imaging, object detection, deep learning, cross-spectral data annotation, multi-sensor imaging system

## Abstract

SWIR imaging bears considerable advantages over visible-light (color) and thermal images in certain challenging propagation conditions. Thus, the SWIR imaging channel is frequently used in multi-spectral imaging systems (MSIS) for long-range surveillance in combination with color and thermal imaging to improve the probability of correct operation in various day, night and climate conditions. Integration of deep-learning (DL)-based real-time object detection in MSIS enables an increase in efficient utilization for complex long-range surveillance solutions such as border or critical assets control. Unfortunately, a lack of datasets for DL-based object detection models training for the SWIR channel limits their performance. To overcome this, by using the MSIS setting we propose a new cross-spectral automatic data annotation methodology for SWIR channel training dataset creation, in which the visible-light channel provides a source for detecting object types and bounding boxes which are then transformed to the SWIR channel. A mathematical image transformation that overcomes differences between the SWIR and color channel and their image distortion effects for various magnifications are explained in detail. With the proposed cross-spectral methodology, the goal of the paper is to improve object detection in SWIR images captured in challenging outdoor scenes. Experimental tests for two object types (cars and persons) using a state-of-the-art YOLOX model demonstrate that retraining with the proposed automatic cross-spectrally created SWIR image dataset significantly improves average detection precision. We achieved excellent improvements in detection performance in various variants of the YOLOX model (nano, tiny and x).

## 1. Introduction

Numerous surveillance imaging systems for outdoor environments used in many military and civilian applications have been developed over the past decades [1]. Surveillance imaging systems are usually based on visible-light cameras. In addition to visible-light cameras, thermal cameras are most often used for night imaging [2,3,4,5].

However, in conditions of smoke or fog, visible-light and thermal cameras provide relatively limited information. In these challenging conditions, due to its advantages (reduced scattering effects and spectral signatures), short-wave infrared (SWIR) cameras provide imagery richer in detail and present the possibility of effective viewing [6,7].

Automatic detection and identification of objects during poor weather conditions (haze, light fog) can be performed using a SWIR camera and an object detection model trained on SWIR images. However, to the best of our knowledge, the current literature lacks information on high performance SWIR detection models. The main prerequisite for training such models is an adequate high-quality training dataset [1,8] which should provide sufficient generalization of the object detection model for various situations which may occur in real-life surveillance applications. Hence, the high-quality training dataset needs to contain a large number of images with objects of interest, which should be captured from different angles, various distances from the camera, backgrounds, weather and lighting conditions, etc. The most common case of creating a training dataset for a specific problem is combining existing publicly available datasets with images recorded in conditions and scenarios that best fit the purpose of the application. However, to the best of our knowledge, there are no large publicly available labeled SWIR image datasets of people and automobiles.

Manually labeling a large dataset is a tedious and time-consuming job. In order to avoid manual labeling and to obtain a large SWIR image dataset, which can be used to train a high-performance SWIR object detector, this paper aims to take advantage of a multi-sensor camera system and transfer learning. The paper proposes a method for automatic cross-spectral annotation of SWIR images using a long-range multi-sensor surveillance system and a deep learning model for the detection of people and automobiles.

After performing exhaustive performance comparisons of different object detection models pretrained on visible-light images (SSD [9], Mask RCNN [10], YOLOv3 [11], YOLOv4 [12], YOLOv5 [13] and YOLOX [14]), we selected YOLOX model to be used in the proposed automatic cross-spectral annotation method, and as a starting point for training our final high-performance SWIR object detection models. The proposed cross-spectral experimental workflow with all the technical and mathematical explanations have been presented in the paper. Using the newly acquired high-quality SWIR dataset we achieve the ultimate goal of training new models based on YOLOX, including *nano*, *tiny* and *x*, which significantly improve detection of people and cars in SWIR images in terms of the average precision, compared to the original models trained on color images. Hence, the new SWIR object detection models can be efficiently used for object detection in conditions when SWIR cameras show significant advantages over the visible-light ones, such as conditions of haze or light fog.

In recent literature, cross-spectral annotation has been used for various applications. Cross-spectral annotation was used in [15] for automatic annotation of the low-resolution far-infrared (LFIR) image human pose estimation dataset. A color camera and human pose estimation model trained on color images are used in combination with an LFIR camera for automatic dataset annotation, where a person is recorded with both sensors in parallel, and an estimate of the human pose on the color image is mapped to the infrared image. In [16], a cross-spectral evaluation dataset, named EDGE20, for multiple surveillance problems was presented. The dataset is used in the problems of pedestrian and facial detection, as well as facial recognition in images captured using visible-light and near-infrared (NIR) cameras. Images were recorded in an outdoor environment under unconstrained conditions during both day and nighttime, followed by a rich set of annotations including person and facial bounding boxes. Paper [17] proposes a framework for cross-spectral pedestrian detection in an unpaired setting. Authors have designed the separate feature embedding networks for color and thermal images followed by sharing detection networks using the assumption that color and thermal image features share characteristics in a common feature space. Moreover, for additional improvement of the cross-spectral feature representation, an adversarial learning scheme was applied to intermediate color and thermal image features.

The remainder of this paper is organized as follows: Section 2 presents SWIR sensor characteristics and advantages compared to visible-light sensors. Section 3 reviews the related work regarding object detection in SWIR images. The experiment workflow with cross-spectral data annotation method is provided in Section 4. In Section 5, we describe SWIR image dataset creation and object detection experiments, present the results and provide a detailed discussion. Section 6 presents the conclusion and a direction for future research in this area. An outline of YOLO-based object detection algorithms is given in Appendix A.

## 2. SWIR Sensor Characteristics and Advantages Compared to Visible (VIS)

SWIR is the name used for a spectral region of the electromagnetic spectrum in the wavelength range of 1 to 3 microns. In this spectral region, similar to the visible range, light is predominantly reflected from objects. Due to its advantages (reduced scattering effects and spectral signatures), SWIR is used in many military and civilian applications. We will present some of the research results that explain the advantages in more detail. In the next sections, we will confirm these findings in an area of interest in multi-sensor imaging systems by presenting imagery in selected scenarios.

Meteorological studies of propagation through hazy and foggy air, originating from the 1950′s [18], set experimentally, proved theoretical expectations regarding the transmission of electromagnetic energy belonging to the optical and infrared regions. Referred methodology for calculation of the spectrophotometric absorption uses optical density per kilometer, quantity dependent of the radius and number of drops contained in the unit volume of the atmosphere. For haze, this value is less than 2 (smaller and fewer droplets), and for stable fog this value rises higher, up to 30. The general conclusion was that in the case of haze, the wavelength for maximum density varies between 0.4 and 0.55 microns; densities decrease rapidly with increasing wavelength. In this scenario, transmission in SWIR spectral region is much higher than in VIS. In the case of fog, results showed that the transmission in the SWIR was not higher than in the VIS.

In more recent literature [7], other advantages of SWIR vs. VIS are referred, e.g., in the case of calculated atmospheric transmission in different climates, by using a MODTRAN atmosphere model and camouflage detection/identification based on reflectivity contrast measurements. All advantages are highlighted in the comparative Table 1. As it can be seen, SWIR has advantages over VIS in multiple categories, from which, in the case of our multi-sensor imaging system for surveillance applications, the most important are: maritime haze penetration, atmospheric transmission, maritime and ground target contrast, turbulence and long-range identification.

**Figure 1:**
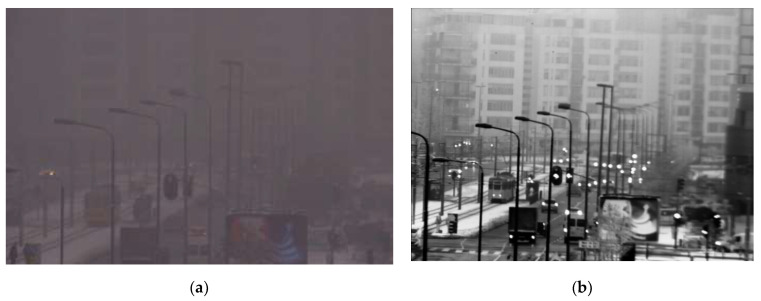
Image of a crossroad at distance of 1 km: (**a**) VIS, (**b**) SWIR.

Experimental field tests with Vlatacom Multi Sensor Imaging Systems (vMSIS) [19] confirmed the presented advantages of SWIR imagers over VIS. Here, we present some examples of images taken by different systems confirming the referred results. In Figure 1, images from VIS and SWIR camera show a scene with a crossroad at distance of 1 km.

**Figure 2:**
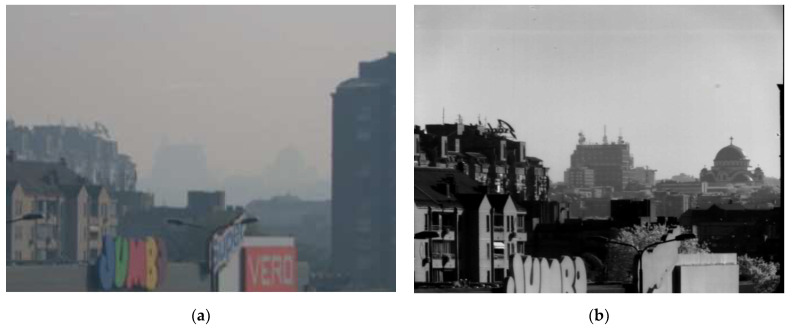
Image of a building at a distance of 4.3 km: (**a**) VIS, (**b**) SWIR.

In Figure 2, images from VIS and SWIR camera show a building at distance of 4.3 km.

In both examples, SWIR images are richer in detail, similar to the corresponding grayscale images obtained using a daylight camera in good weather conditions. From this point, it seems that training with images taken in SWIR can improve the automatic detection of the objects during poor weather conditions (haze, light fog).

The current situation on the market of SWIR cameras for commercial applications shows that Focal Plane Array (FPA) detectors in InGaAs technology, which does not require cryogenic cooling and can function at room temperature are the most popular choice in modern systems. Technology for these detectors can enable resolution of 10 μm pixel pitch, with formats up to 1280 × 1024 pixels [20].

Special attention is paid to pixel Read Out Integrated Circuitry (ROIC) in SWIR camera developments. According to the applications of the SWIR camera, ROIC can be optimized, so that SWIR sensors can perform best. In many applications, the use of wide dynamic logarithmic ROIC can be helpful [21], which is of great importance in low light conditions, when glare from street and car lights saturate the image of the linear ROIC.

## 3. Related Work in SWIR Object Detection

In [22,23] SWIR camera was used within the unmanned ground vehicle (UGV) together with sensors in other wavebands (MWIR, LWIR) for analyzing soil and detection and localization of mud in the world model which UGV can use to plan safe trajectories. In [24], the authors introduced the WVU Outdoor SWIR Gait (WOSG) dataset built with a SWIR camera to evaluate the performance of gait recognition algorithms in SWIR videos. The evaluated algorithms do not require a large dataset and this dataset contains 155 subjects and presents gait information acquired in an uncontrolled, outdoor environment. Three gait recognition algorithms on the created SWIR dataset are evaluated, with special emphasis on the design of algorithms for segmentation and recognition of individuals in the SWIR domain due to significant operational advantages in contrast to color images. The authors of [25] presented an active SWIR imaging system for the detection, tracking, and identification of human targets at ranges of several hundred meters. A system was developed with the possibility of generating recognizable facial images, day or nighttime, with recognition capabilities achieved at distances up to 350 m. Paper [26] deals with pedestrian detection in SWIR images under different visibility conditions—clear sky, haze, and fog. A classical approach based on the histogram of oriented gradients (HOG) features and a support vector machine (SVM) classifier based on deformable part models and trained on a PASCAL dataset of images in the visible light spectrum, was applied for pedestrian detection. The same approach using SVM classifier and HOG features was applied in [27], where the SVM classifier was trained on features obtained from visible light images of an INRIA dataset, while tests were conducted in clear visibility conditions. In [28] active-SWIR Tactical Imager for Night/Day Extended-Range Surveillance (TINDERS) systems used for detection and tracking of a person while walking and automated face recognition in daytime and nighttime operation, and at various distances ranging from 100 m to 350 m. The pedestrian detection system on Near Infrared images is presented in [29]. It consists of three modules, each based on Speeded-Up Robust Features (SURF) matching. The first generates regions of interest (ROI), primarily based on a high recall rate with the hierarchical codebook of SURF features located in the lighter area of the head regions. The second uses an SVM to classify the whole body, and the third combines the mean shift algorithm inter-frame scale-invariant SURF feature tracking to improve the robustness of the whole system. In [30] authors analyzed vehicle detection in visible-near infrared-short-wave infrared (VNIR-SWIR) images based on spectra using a method that combining SVM classifier and data processing (normalized reflectance data, an optional non-diagonal whitening transform). The targets included multiple vehicles with silver paint, excluding vehicles with very dark paint, other than green cars. In [31] low quality SWIR videos were used to perform vehicle tracking directly in the compressive measurement domain using videos with missing data. They used the ResNet-18 (18-layer convolutional neural network) that has the advantage of avoiding performance saturation and/or degradation when training deeper layers and built a two classes model (background and vehicle) using about 150,000 vehicle images and about 500,000 background images. Authors showed that the deep learning tracker performs better than conventional trackers and suggested new enhancements and future research directions. The authors improved car tracking performance in [32,33,34] including YOLO model version 3 [11] for target tracking. The classification is still performed using ResNet due to better classification than YOLO in the compressive measurement domain for SWIR videos. In [35] a multi-sensor camera system consisting of SWIR, thermal and hyperspectral camera was developed for monitoring and object detection. Regarding object detection, a detector-agnostic procedure was developed, integrating both motion detection techniques (background subtraction—BS) and deep convolutional neural networks. Based on the object detectors (BS, Faster R-CNN [36], and YOLOv2), the possible detected targets extracted from the thermal and SWIR sensors are then projected into the local coordinate system using the transformation matrix, and the resulting coordinates are then projected to the hyperspectral image plane using the inverse transformation matrix. These projected targets then directly fuse the spectral information from the hyperspectral bands. Overall accuracy results on the dataset presented in the paper in challenging indoor and outdoor scenes are reported at 43%, 66%, 59% for the BS, Faster R-CNN, and YOLOv2 methods, respectively. In [37] an autonomous platform for data collection and algorithm testing for land autonomous vehicles, with a computer vision hardware complex containing four SWIR cameras, is presented. The platform is also supported with 10 short-focus and two long-focus machine vision camera lidars, and as such has the possibility to collect video and three-dimensional data and try out algorithms for three-dimensional reconstruction, semantic segmentation, and obstacle classification.

## 4. Experiment Workflow

### 4.1. Problem Formulation

The aim of the experiment is to detect people and cars in urban scenes in different weather conditions, times of day, and recording conditions using SWIR sensor and a real-time object detector.

As the creation of a dataset of SWIR images is based on the use of the parallel recording in the visible-light and SWIR channels, the automatic creation of a dataset requires a detector with good performance on the color images. By analyzing and comparing state-of-the-art detectors: SSD [9], Mask R-CNN [10], YOLOv3 [11], YOLOv4 [12], YOLOv5 [13] and YOLOX [14], a YOLOX detector was chosen for cross-spectral SWIR dataset creation.

Although the objects in SWIR images differ from those in color images in both color and details, especially for images taken with an interlaced camera format, these objects still have sufficiently similar characteristics to those in color images, thus models trained on the color image dataset are expected to bear sufficient generalization to detect objects on SWIR images. As such these models can be taken as a basis for training models for the SWIR image channel.

To train the object detection model, we needed an appropriate database of SWIR images of urban scenes. To the best of our knowledge, there is no adequate publicly available database of labeled SWIR images, thus we propose a cross-spectral method which uses a multi-sensor imaging system to create our database of SWIR images of urban scenes in different recording conditions to train the object detection model.

### 4.2. Multi-Sensor Imaging System

**Figure 3:**
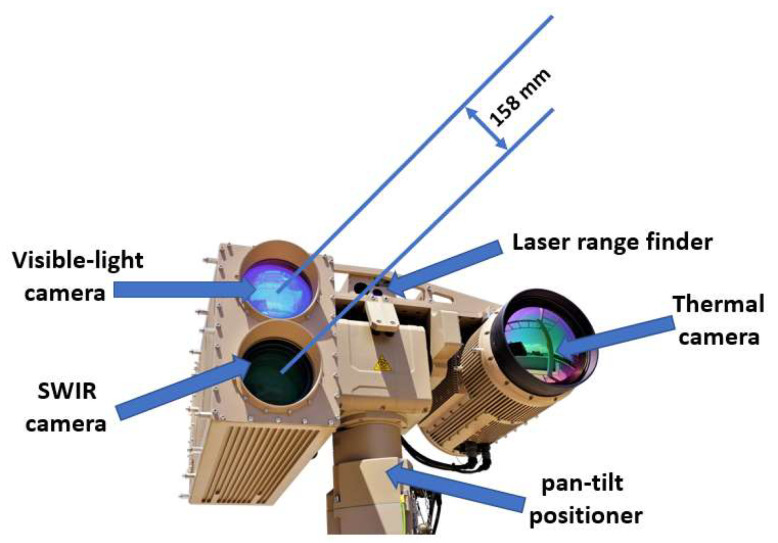
Vlatacom Multi Sensor Imaging System: vMSIS3-CSD-C825-T.

For the purpose of creating a SWIR dataset, Vlatacom Multi Sensor Imaging System vMSIS3-CSD-C825-T model is used—Figure 3. System vMSIS3 is a complex system that has three high resolution imaging channels (visible-light, SWIR and thermal) and an optional laser range finder, Global Positioning System (GPS) and digital magnetic compass (DMC) components. The cameras are placed on the positioning platform—pan-tilt. The pan-tilt positioner is a two-axis servo-driven device designed for long-range video systems that requires azimuth and elevation rotation with high accuracy and high angular velocity. This allows rapid setup of the system for recording different scenes.

As the SWIR and visible-light cameras are required for the analysis in this paper, Table 2 presents the characteristics of the SWIR and visible-light (low-light) cameras in system vMSIS3-CSD-C825-T.

**Figure 4:**
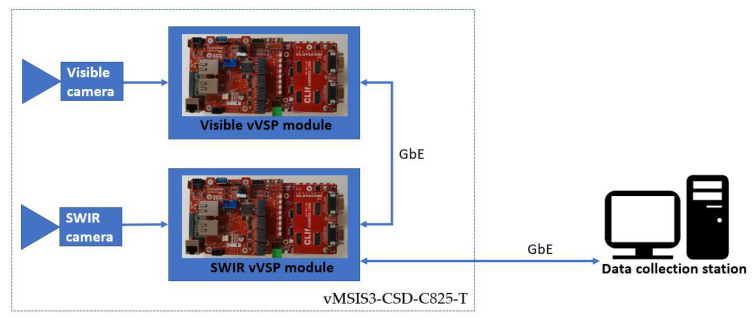
Block diagram of the connecting cameras to data collection station.

Each camera is connected with a Vlatacom Video Signal Processing (vVSP) platform [38] via appropriate interface board. The vVSP can capture and process video signals from various types of cameras and provide outputs as IP stream. In addition, vVSP unit performs system control. Control and video data acquisition is provided by software components in different layers that provide connectivity of the system to the standard interfaces. Each vVSP module has an integrated Gigabit Ethernet (GbE) switch, so that the modules in the vMSIS3 system are interconnected via GbE. The vMSIS3 system provides one GbE output to the outside world. Data collection station accesses the systems from the console application which has the possibility of controlling the device, to reproduce and record streams from vMSIS3 cameras. Block diagram of the system is shown in Figure 4.

### 4.3. Cross-Spectral Data Annotation

**Figure 5:**
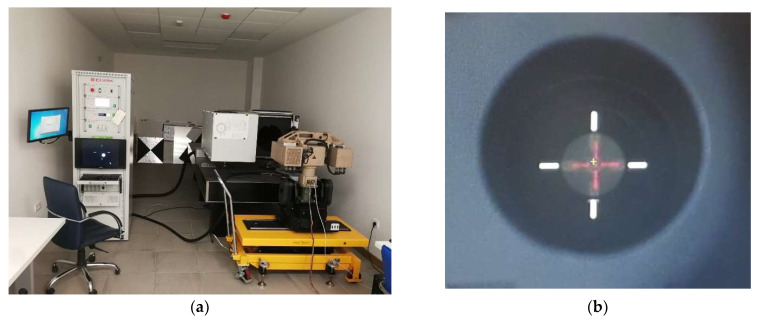
Calibration procedure in Vlatacom electro-optical laboratory (**a**) and alignment of camera axes and laser beam (**b**).

With the aim of providing images of the same scene for parallel recording, optical axes of the cameras in the multi-sensor imaging system must be aligned. This is achieved during boresight calibration procedure [39] of the multi-sensor imaging system in Vlatacom electro-optical laboratory—Figure 5. Mechanics of the system are designed to allow fine tuning of the cameras position in reference to a laser range finder that is an additional element in the multi-sensor imaging system. For boresight calibration, an image of crosshair target is projected using collimator and integration sphere, to provide a reference for optical axis of each camera to be aligned with the laser, as presented in Figure 5. Precision of the alignment is better than divergence of the laser beam which is 0.7 mRad.

Although the field of view (FOV) is adjustable, SWIR and visible-light camera FOVs cannot be set to precisely the same values. The SWIR camera has a wider vertical FOV, while the visible-light camera has a wider horizontal FOV.

In order to successfully transfer detections from one camera image to another, before collecting the data for annotation, it is necessary to set the same FOV for both cameras. Mapping detections from visible-light channel to SWIR channel is possible only in the overlap zone between visible-light and SWIR FOV. In order to create a high-quality ground-truth dataset, without missed detections, the SWIR images were cropped to the overlap zone.

**Figure 6:**
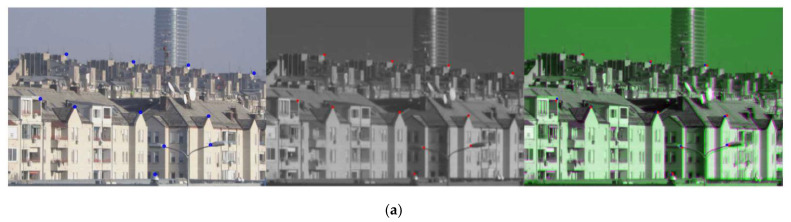

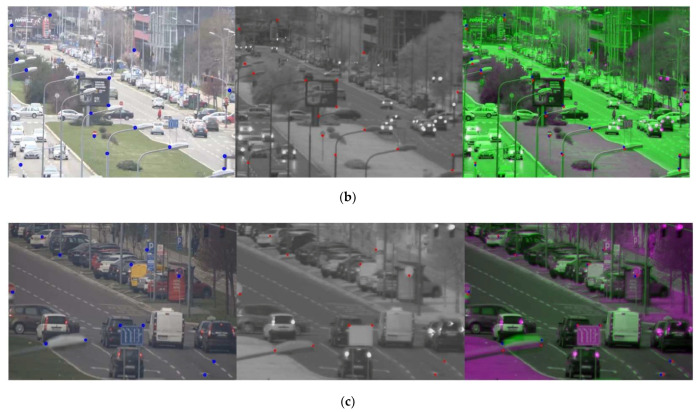
Visible-light image, SWIR image and fused image of the scene taken with: (**a**) the least common FOV of 6.127 deg, the distance to the nearest object in the image is 420 m, and to the farthest is 2650 m; (**b**) FOV of 6.32 deg, the distance to the nearest object in the image is 280 m, and to the farthest is 1735 m; (**c**) FOV of 1.67 deg, the distance to the nearest object (street lamp) in the image is 255 m, and to the farthest is 810 m.

Despite retaining only the part of the image in the overlap zone, distortions between visible-light and SWIR images are still present. In Figure 6, examples are shown of images cropped in the overlap zone, resized to the same size (visible-light image is resized to the SWIR image size) and fused without blending to make the discrepancies more noticeable. Moreover, a set of points on static objects are chosen to more clearly demonstrate the imperfection of the matching between the SWIR and the visible-light image.

In order to accurately transfer the detections from the color image to the SWIR image in the overlap zone, and due to the distortion problems, a geometric correspondence between the SWIR and the color image needs to be established. The first step in the processing pipeline is to calculate the transformation matrix between the images—the homography matrix.

For a fully automatic process of mapping detections, discovery of corresponding points between visible-light and SWIR images using certain feature detectors (e.g., Harris Corners, SURF, HOG) is needed. However, feature-based determination of corresponding points is extremely difficult and unreliable due to the different captured wavelength range and interlaced nature of the used SWIR camera. Therefore, we determined the homography matrix based on manually selected corresponding points on both images (only on the first frames of the SWIR and visible-light video sequences).

We used non-reflective similarity transformation [40]. This transformation is used in the case when shapes in the corresponding images are unchanged, but distortion by some combination of translation, rotation, and scaling is present. The transformation has four degrees of freedom and needs only two pairs of corresponding points. It supports rotation, translation and isotropic scaling [40]. Non-reflective similarity transformation is expressed with the matrix *H*:

(1) $$H=\left[\begin{array}{c} h_{1}\\ h_{2}\\ h_{3} \end{array}\;\;\begin{array}{c} -h_{2}\\ \;\;h_{1}\\ \;\;h_{4} \end{array}\right].$$

The projection ($\hat{x}$, $\hat{y}$) of the image point (*x*, *y*) by matrix *H* is given by:

(2) $$\left[\begin{array}{cc} \hat{x} & \hat{y} \end{array}\right]=\left[\begin{array}{ccc} x & y & 1 \end{array}\right]\;H.$$

After non-reflective similarity transformation, straight lines remain straight, and parallel lines remain parallel.

After the homography matrix is calculated, the mapping of the detections from the visible-light image is performed on the transformed SWIR image. As images in the original domain (not transformed) are needed to train the detector, the detections must be returned to the original domain by inverse transformation. The advantage of this approach is that it does not require the actual transformation of the entire visible-light and SWIR images, which is an expensive computational process. Only the coordinates of the detected objects in the visible-light image are converted between the reference systems.

Moreover, there are many moving objects in the scene that are recorded to create a dataset. The described homography transformation is independent of moving objects in the scene. Although a precise transformation between an SWIR and a visible-light image was performed, it does not guarantee accurate mapping of moving object detection if the cameras are not well synchronized. The SWIR camera works with the frame rate of $f_{s}$, while the visible-light camera works with the frame rate of $f_{v}$. To perform the mapping between the corresponding pairs of images in the video sequence, which is also accurate for moving objects, a precise synchronization between the cameras is required. As the correlation between successive frames in the video sequence is very high, not every frame from the video sequence must be used to create a dataset. The object detection in a visible-light image and detections mapping to the SWIR image are performed between pairs of images defined with:

(3) $$N_{s}=N_{v}\frac{f_{s}}{f_{v}},$$

where $N_{s}$ is frame number in SWIR video sequence, while $N_{v}$ is frame number in visible-light camera video sequence.

**Figure 7:**
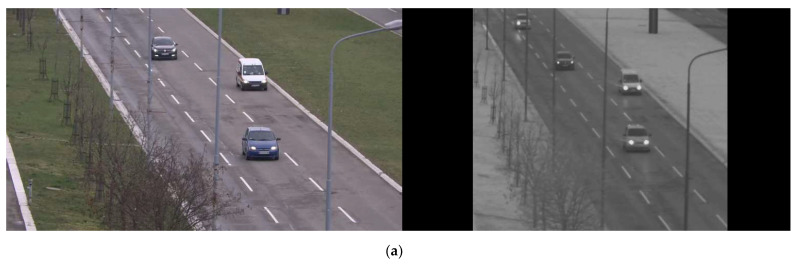

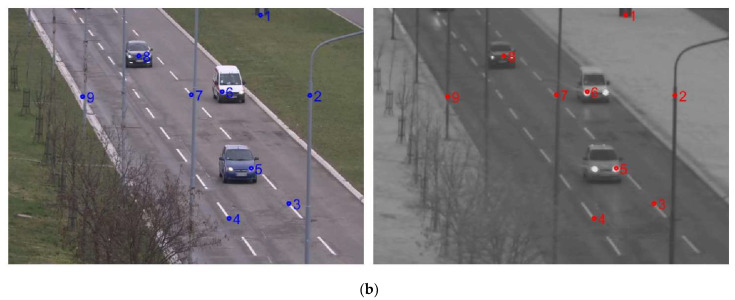
(**a**) Visible-light image (left) and SWIR image (right) of the same scene taken with the Vlatacom Multi-Sensor Imaging System (vMSIS3). (**b**) Images after synchronization and cropped in the overlap zone. Red points on the SWIR image represent the corresponding blue points mapped from the visible-light image.

SWIR and visible-light images from Figure 7a after synchronization and cropped in the overlap zone are shown in Figure 7b. Each detection is presented with a bounding box defined with two points (top left corner and bottom right corner), thus only these points need to be mapped. The result of the mapping procedure is shown in Figure 7b, where the red points in the SWIR image represent projections of the corresponding blue points from the visible-light image on static as well as on moving objects.

### 4.4. Selection of Object Detection Models for Visible-Light Channel

The creation of a SWIR dataset is based on mapping the detections from parallel recorded video sequences with a visible-light camera. In order to exhibit as much as possible accurate mapping, it is necessary for the visible-light image object detector to be as effective as possible, with as few false and missed detections as possible. In order to evaluate some of the state-of-the-art models for object detection in color images that would form part of an overall algorithm for detection mapping and automatic creation of SWIR dataset, we created a custom dataset of color images. The dataset is very challenging regarding the images illustrating realistic scenes in which a long-range surveillance system is applied. As the classes of interest for detection are people and cars; this dataset contains images of urban scenes with many people and cars in them. The dataset contains 1160 color images, manually labeled for classes of people and cars. Yolo mark [41] is used for labeling, which provides the possibility of very fast manual labeling.

Without changing the original architecture, SSD, Mask RCNN, YOLOv3, YOLOv4, YOLOv5 and YOLOX models are used for analysis. In this step, only the performance of the model is of interest, not the processing time, because this is the part of the algorithm that works offline. The COCO_eval library [42] was used for evaluation. The evaluation was performed on the image resolution of 640 × 640.

Comparative results of the evaluated detectors are given in Table 3. Based on the presented results, the YOLOX model was decided to be an integral part of the algorithm for the automatic creation of the SWIR dataset.

## 5. Results and Discussion

### 5.1. Detection Mapping from Visible-Light to SWIR Channel

**Figure 8:**
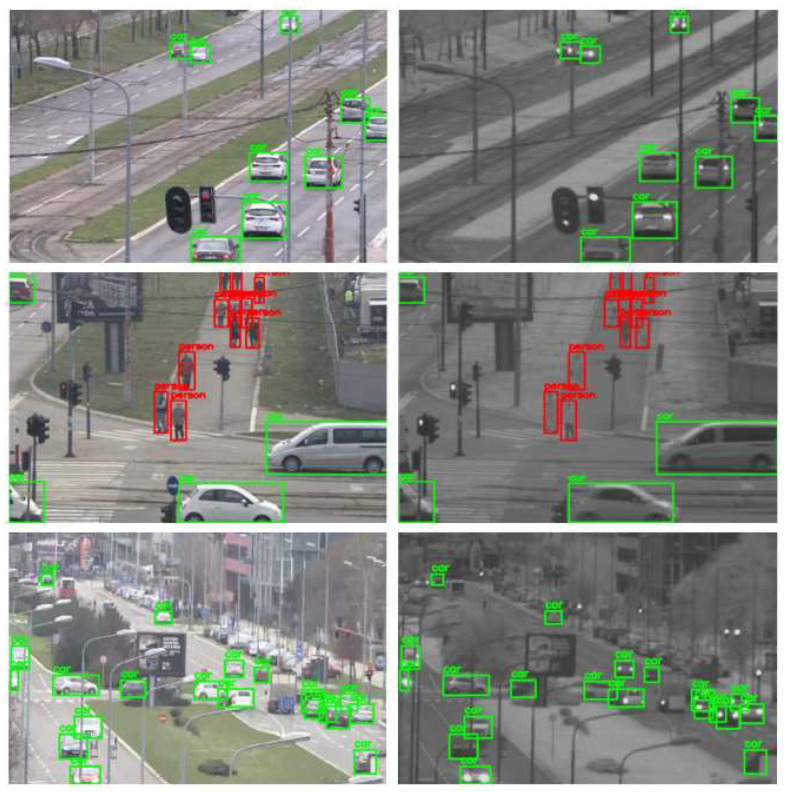
Results of mapping detections (car—green, person—red) from visible-light (**left**) to SWIR (**right**) images using YOLOX-x model for the detection and mapping method explained in Section 4.3.

For the evaluation of the proposed cross-spectral data annotation method, we created a dataset of 1339 SWIR images with different resolutions depending on FOV and overlap zone between cameras. The dataset is created using a SWIR and color camera in the parallel recording of different urban scenes at different fields of view, creating 20 video sequences. As detection mapping is only possible in the overlap zone between cameras, this dataset contains only images cropped in the overlap zone, so that the resolution of these images is dependent on the FOV: 465 × 340, 519 × 369, 519 × 392, 521 × 391, 522 × 360, 533 × 380, 536 × 391, 541 × 392, 555 × 371, 559 × 350, 573 × 398, 575 × 412, 576 × 276, 576 × 389, 576 × 411, 576 × 414, 576 × 415, 576 × 419, 576 × 420, 576 × 423. All cropped SWIR images are manually labeled using Yolo_mark [41] for person and car class. Several examples of images from the dataset with the mapping results are shown in Figure 8.

Detection mapping is performed using the YOLOX-x model [14] with an image input resolution of 640 × 640 pixels. The detection acceptance threshold on the color channel is set to 0.3. The obtained detections on the color image are then scaled to the resolution of the SWIR image and transformed using the calculated homography matrix. By applying the inverse transformation, the detections are transferred back to the original domain of the SWIR image. The results of the algorithm for detection mapping from color to SWIR images are shown in Table 4. The results were obtained using the COCO_eval library [42].

**Figure 9:**
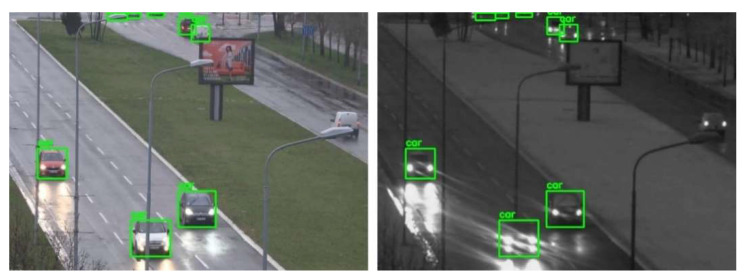
Mapping detections (car—green, person—red) from visible-light (**left**) to SWIR (**right**) image and missed car detection due to the partial occlusion of the streetlamp.

**Figure 10:**
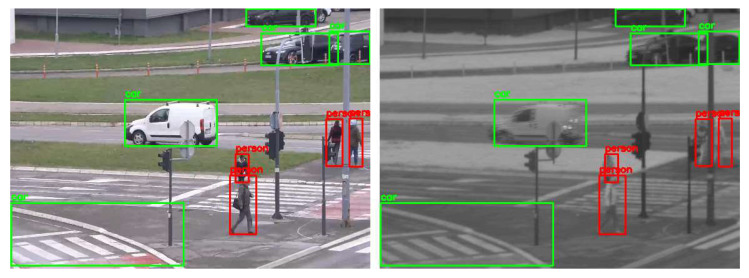
An example of incorrect car detection mapping due to false detection on the color image.

**Figure 11:**
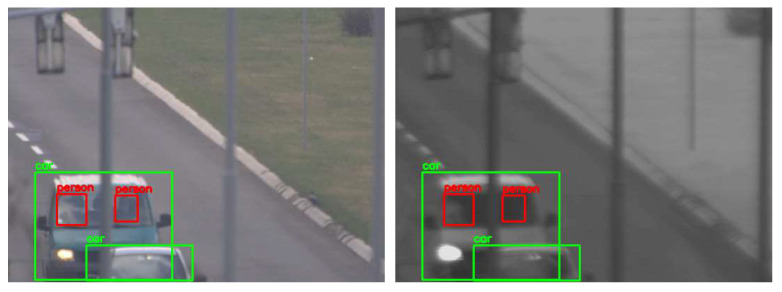
An example of people detection mapping from color to SWIR image, although people in the car are not clearly noticeable in the SWIR image due to the nature of the SWIR sensor.

**Figure 12:**
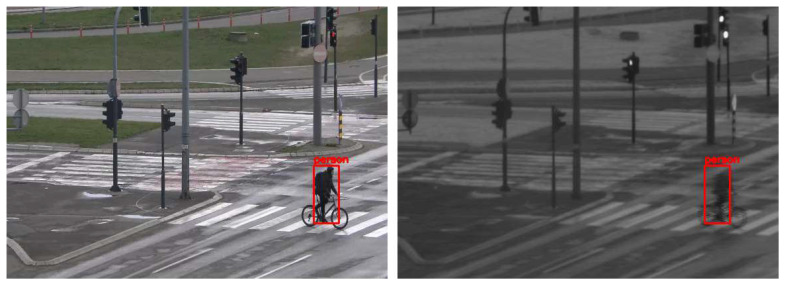
Inaccurately placed bounding box around a person in the SWIR image due to the detection of a moving person, and the method the image is created using an interlaced camera.

Based on the presented results in Table 4, it can be seen that the method exhibits excellent performance, where all correctly detected objects by YOLOX are transformed and mapped in the correct way. The presented results exhibit that a high-quality dataset can be created automatically by the method of cross-spectral mapping with minimal manual effort. However, upon analyzing the obtained results, it was concluded that there are several reasons for not achieving the maximum precision in the detection mapping:

1. Missed detections on the color image—Figure 9. Due to partial occlusions, some objects remain undetected on the color image, although they are significantly visible and should have been part of the ground-truth SWIR dataset.
2. False detections on the color image—Figure 10.
3. Detections of objects on the color image that are not visible on the SWIR image due to the nature of the SWIR sensor and should not be part of the SWIR training dataset—Figure 11.
4. Poorer precision in detection mapping of moving objects, especially if they are moving at a higher speed, due to the nature of the image on the SWIR camera. The used SWIR camera is an interlaced camera, thus there is inaccuracy in detection mapping from the color image because the position of the object on the SWIR image at the time of sampling is not unambiguously determined. An example is shown in Figure 12.

### 5.2. Selection of the Object Detection Model for Development of SWIR Object Detection

In order to select the neural network architecture to be trained on SWIR images, the performance of state-of-the-art object detection models trained on color images from COCO dataset [43] were tested on our custom SWIR test dataset. SSD using backbone ResNet-50, Mask RCNN—backbone ResNet-50, YOLOv3—backbone Darknet-53, YOLOv4—backbone CSPDarknet-53, YOLOv5—backbone CSPDarknet-53 and YOLOX-x—backbone Modified CSPv5 were used for testing. YOLOv3 and YOLOv4 are implemented and tested in Darknet framework, while SSD, Mask RCNN, YOLOv5, and YOLOX are implemented and tested in PyTorch framework. The testing experiment was performed on our manually labeled test SWIR dataset containing 715 full resolution SWIR images and comparative results are presented in Table 5. Moreover, Table 5 provides interference time for each model. All the models are tested at 640 × 640 resolution on an NVIDIA GeForce RTX 2080 GPU using CUDA acceleration library. The obtained results on SWIR test dataset (Table 5) suggest that an additional training set is needed to obtain optimal performance, which motivates our work on automatic cross-spectral data annotation to obtain adequate SWIR datasets.

As observed in the case regarding the experiment of testing these models on our custom-created color image dataset in Section 4.4, the YOLOX model pretrained on color images had significantly better results in object detection on SWIR images compared to other tested models. YOLOv3-v5 models are anchor-based models and to achieve optimal detection performance, clustering analysis is needed to determine a set of optimal anchors before training. Another drawback is that clustered anchors are domain-specific, less generalized and with anchor mechanism the complexity of detection heads is increased. With an anchor-free mechanism [14], the YOLOX training and decoding phase are simpler, and the number of design parameters is reduced. However, YOLOX exhibits the highest inference time compared to others tested models, as it can be seen in Table 5. Taking this into account, YOLOX achieves a better trade-off between training complexity, accuracy, and speed than other tested models. Therefore, we have selected the YOLOX model for SWIR object detection model development and further training using the proposed cross-spectral training dataset creation methodology.

### 5.3. Improved SWIR Object Detection by New Methodology Based on Cross-Spectral Data Annotaion

Dataset for the YOLOX model training was created using the cross-spectral annotation method presented in Section 4.3. The object detector used for the color images is the YOLOX-x model, with a resolution of the input image of 640 × 640 pixels, and the threshold for accepting and mapping the detection set to 0.3. The sampling rate is one frame per second to reduce the correlation between the images in the dataset. SWIR images cropped in the zone of overlapping between SWIR and visible-light camera’s FOV entered the SWIR dataset. The dataset is divided into a training and a validation part, with 10,023 images included in the training dataset and 717 images in the validation set. The training set contains images of urban scenes, and people and cars are objects of interest. Recorded scenes present a real area under surveillance, where people are at different distances from the camera and move with different body orientations and movement speeds. Cars are in motion on the streets but also parked in parking lots.

As suggested in [14], the training was performed for 300 epochs with 5 epochs warmup, stochastic gradient descent with an initial learning rate of 0.01 and cosine learning rate schedule, momentum 0.9, and weight decay 0.0005. During the training of the YOLOX-x, mix up and the mosaic data augmentation with the scale range [0.1, 2.0] was used, while for training smaller models YOLOX-Nano and YOLOX-Tiny the mix up augmentation was removed and the mosaic scale range was set to [0.5, 1.5]. The input image size was 640 × 640. Training was operated on a PC with an i7-8700K 3.70 GHz CPU, 32GB RAM, and an NVIDIA GeForce RTX 2080 GPU. The batch size was set to 2 due to the capacity of the used GPU.

Table 6 presents the comparison of the detection results of the original YOLOX-based models (trained on color COCO image dataset) and the YOLOX-based models trained on our automatic cross-spectrally created SWIR dataset. To the best of our knowledge, there are no publicly available labeled SWIR image datasets of people and cars on which the models can be tested and compared, thus models were tested on our manually labeled test SWIR dataset containing 715 full resolution SWIR images (which is the same as in Section 5.2). The results clearly indicate an improvement in the performance of the model trained on the SWIR dataset. For a fair comparison, to show only the impact of the automatically created SWIR image dataset by cross-spectral methodology on model performance, all models are trained under the same conditions as the original. This shows that the automatically generated dataset by the cross-spectral methodology, even with the explained shortcomings, significantly improves the detection performance on the SWIR channel.

Although pretrained YOLOX-x shows the best detection performance on the SWIR channel, it also requires the highest inference time. As fewer complex models are more suitable for implementation on EDGE platforms such as vVSP, we have also trained YOLOX-Nano and YOLOX-Tiny with the proposed methodology. Inference time for YOLOX-Nano model was 22.815 ms, YOLOX-Tiny 23.283 ms and YOLOX-x 87.294 ms (measured at 640 × 640 resolution on NVIDIA GeForce RTX 2080 GPU using CUDA acceleration library). Results presented in Table 6 show that all three models trained on SWIR images obtained with proposed cross-spectral methodology in this paper significantly improved average precision detection performance (YOLOX-Nano: 39.5%, YOLOX-Nano: 33.9%, YOLOX-x: 10.3%) for cars and persons classes. Moreover, all models improved detection performance for these classes separately, as well as for all sizes of these objects separately. Improvements of the models trained on the SWIR dataset created by our cross-spectral methodology under good weather conditions can be efficiently utilized for object detection in poor weather conditions (such as haze or light fog) in which only the SWIR camera provides the possibility of effective detection and provides an image that is very similar to images in good weather conditions, as shown in Figure 1 and Figure 2.

The only case of reduced performance is seen in the detection of large objects (size greater than 96 × 96 (9216) pixels) by the YOLOX-x model. The reason for this can be seen in the percentage of large objects in the training dataset. The created training set with the proposed methodology contains 58,605 objects, of which 5.94% are large-sized, 59.95% are medium-sized and 34.11% are small-sized. However, for application in long-range surveillance system, the detection of small objects is of greater importance, because in the images of scenes from a few kilometers away from the system, the objects are usually up to 32 × 32 pixels.

## 6. Conclusions

This paper deals with object detection in SWIR spectral domain using a deep learning method which was shown to be very successful for object detection in visible-light spectrum and long-range multi-sensor surveillance systems. Based on the preliminary examination and comparison of the selected state-of-the-art object detection models such as SSD, Mask RCNN, YOLOv3, YOLOv4, YOLOv5, and YOLOX which achieve very good detection performance in the visible-light spectrum, YOLOX model was used as the baseline for further experiments. In particular, the paper was focused on people and car detection in urban areas using SWIR camera. As there are no large publicly available labeled SWIR image datasets of people and cars, the paper proposes a method for automatically creating and annotating a high-quality SWIR image dataset. The method is based on cross-spectral mapping of detection. For a scene recorded with visible-light and SWIR camera in parallel, a YOLOX model trained on color images was used to detect people and cars in color images, and then by applying a set of transformations the detections were mapped to the SWIR images. The cross-spectral detection mapping method exhibits very high accuracy in object detection and mapping detection, and on our manually labeled SWIR dataset for detection mapping evaluation of 1339 images achieves an average precision of 89.2%, namely 83.5% for small objects, 91.3% for medium sized objects and 89.7% for large sized objects. For such an automatically created SWIR training dataset, the experiment shows that the detection performance of the final retrained YOLOX model on SWIR images was significantly improved (as much as by 39.5% for the YOLOX-Nano model).

As the SWIR cameras are especially suitable for operation in conditions of haze and fog, the long-range system with an SWIR camera is widely used in maritime surveillance. For applications in maritime surveillance, our future research will include the automatic cross-spectral creation of ship datasets and the detection of ships using SWIR sensors.

## Figures and Tables

**Table 1:** Summary of band comparison for daytime considerations.

Daytime Considerations	VIS	NIR	SWIR
Maritime haze penetration	bad	moderate	good
Fog penetration	bad	bad	bad
Atmospheric transmission	bad	moderate	good
Cloud penetration	bad	bad	bad
Forrest fire and fog oil penetration	bad	moderate	good
“See” laser designator spot	moderate	moderate	good
Camouflage detection/identification	moderate	moderate	moderate
Urban and rural background contrast	good	good	moderate
Maritime and ground target contrast	moderate	good	good
Skin detection	moderate	moderate	moderate
Spectral discrimination	moderate	moderate	good
Turbulence	moderate	moderate	good
Long-range identification (3 inch aperture soldier)	moderate	moderate	good
Long-range identification (6 inch aperture platform)	bad	moderate	good

**Table 2:** Visible-light and SWIR camera properties.

Camera Properties	Visible	SWIR
Resolution	1920 × 1080	576 × 504
Frame rate	30	25
Minimal FOV [deg]	0.73	0.581
Maximum FOV [deg]	26.61	6.126
Optics	Motorized continuous zoom lens	Motorized continuous zoom lens
Minimal focal length [mm]	12	100
Maximum focal length [mm]	550	1000

**Table 3:** Comparative results of different models for the person and car classes detection on a dataset of color images. Results are presented with Average Precision (AP) 0.5:0.95 using COCO_eval library.

Model	All Objects (Person and Car)
SSD	0.3%
Mask RCNN	23.7%
YOLOv3	24.9%
YOLOv4	21.6%
YOLOv5	20.9%
YOLOX	**27.6**%

**Table 4:** Evaluation of the algorithm for detection mapping. Evaluation is performed on labeled SWIR images using COCO_eval library. Small size object presents objects up to 32 × 32 (1024) pixels, medium: from 32 × 32 (1024) to 96 × 96 (9216) pixels, and large objects with size greater than 96 × 96 (9216) pixels. Performance is presented with the Average Precision (AP) 0.5:0.95.

AP (0.5:0.95)	All Sizes	Small	Medium	Large
All objects	89.2%	83.5%	91.3%	89.7%
Person	83.9%	72.1%	87.1%	100%
Car	94.5%	95.0%	95.6%	89.7%

**Table 5:** Comparative results of different pretrained models on color images for the persons and cars detection together, as well as separately, on the test SWIR dataset. Results are presented with Average Precision (AP) 0.5:0.95 using the COCO_eval library.

Model	All Objects (Person and Car)	Person	Car	Inference Time [ms]
SSD	1.2%	0.4%	2%	5.956
Mask RCNN	28.1%	28.3%	28.0%	85.034
YOLOv3	36.0%	34.8%	37.1%	33.727
YOLOv4	20.4%	19.3%	21.5%	38.686
YOLOv5	28.3%	29.1%	27.5%	53.889
YOLOX	**55.9**%	**48.6**%	**63.2**%	87.294

**Table 6:** Evaluation of the original YOLOX model and our trained YOLOX model on SWIR dataset for people and car detection on SWIR images. Evaluation is performed on labeled SWIR images using the COCO_eval library. Small size object presents objects up to 32 × 32 (1024) pixels, medium: from 32 × 32 (1024) to 96 × 96 (9216) pixels, and large objects with sizes greater than 96 × 96 (9216) pixels. Performance is presented with Average Precision (AP) 0.5:0.95.

AP [%]		All Sizes		Small		Medium		Large	
		Original	Trained on SWIR	Original	Trained on SWIR	Original	Trained on SWIR	Original	Trained on SWIR
YOLOX Nano	All	19.4	**58.9**	8.9	**45.7**	25.0	**64.8**	35.1	**68.2**
	Person	17.5	**53.7**	5.2	**36.6**	22.9	**60.2**	34.0	**58.9**
	Car	21.2	**64.1**	12.7	**54.8**	27.2	**69.4**	36.3	**77.4**
YOLOX Tiny	All	28.4	**62.3**	18.5	**52.2**	33.8	**67.0**	50.1	**73.1**
	Person	25.9	**57.6**	17.1	**45.1**	29.6	**62.3**	50.0	**68.5**
	Car	30.9	**67.1**	19.9	**59.3**	38.1	**71.8**	50.2	**77.6**
YOLOX x	All	55.9	**66.2**	51.0	**57.3**	58.1	**70.3**	**86.5**	67.6
	Person	48.6	**61.8**	43.6	**50.6**	50.0	**66.2**	**93.3**	58.6
	Car	63.2	**70.5**	58.5	**64.1**	65.7	**74.5**	**79.7**	76.7

**Figure A1:**
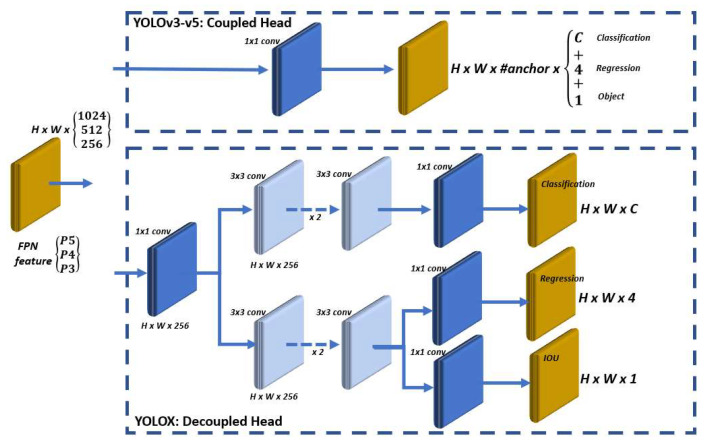
Coupled head in YOLOv3 and decoupled head in YOLOX.

## Appendix A

Computer vision algorithms for object detection can basically be divided into two categories: classification-based and regression-based. For identified regions of interest with a high probability of object detection, classification-based algorithms, classify these regions using Convolutional Neural Networks (CNN). These two-step algorithms are usually very slow and not applicable in real-time. The most well-known two-step classification-based algorithm is RCNN [44] and its improvements Fast-RCNN [45] and Faster-RCNN [36]. Unlike two-step classification-based algorithms, regression-based algorithms process the input image in a single step. The entire image is passed through the neural network and determination of the bounding boxes and classes of the detected objects is performed after only one evaluation of the input image. With this feature, these algorithms work much faster than two-step algorithms and find application in real-time operation. The best-known representatives of this class of algorithms are SDD (Single Shot Detector) [9] and YOLO (You Only Look Once) [46].

In the YOLO algorithm, the input image is first divided into a grid of N x N cells. If the center of an object falls into a grid cell, that grid cell is responsible for detecting that object, while all others ignore it even though parts of the object are in those grid cells. Further, for each grid cell, defined number of bounding boxes are predicted, each of which has an estimate of 4 parameters ((*x*, *y*, *w*, *h*) corresponding to *center coordinate* (*x*, *y*), *width*, *height* of a bounding box) and confidence score. Confidence score in this case refers to the probability that an object exists within each bounding box.

The problem where each grid cell can detect only one object is solved by YOLOv2 using anchor boxes [47]. Anchor boxes represent a list of predefined bounding boxes with certain width and height that best match in size the desired objects. In YOLOv2 number of anchor boxes is determined using k means clustering algorithm on the training set of the ground truth bounding boxes based on intersection over union (IOU) metrics between the bounding box and the centroid. The predicted bounding box is assigned not only to the certain grid cell but also to one of the anchor boxes with the highest IOU with the ground truth bounding box. The problem of bounding boxes which are not objects or that draw more than one bounding box per object is solved with the Non-Maximum Suppression (NMS) method [47]. NMS removes all the overlap bounding boxes which have an IOU value higher than the defined threshold value.

YOLOv3 [11] includes improvements over previous versions in terms of speed, precision and class features. The feature extractor used in YOLOv3 is a hybrid of Darknet-19 used in YOLOv2 and Residual Network (ResNet). The network is built with the bottleneck structure (1 × 1 followed by 3 × 3 convolutional layers) inside each residual block plus a skip connection. This new feature extractor network contains 53 convolutional layers thus that it is called Darknet 53 [11]. YOLOv3 introduces predictions across scales. The features from the last three residual blocks are used for three different scale bounding box predictors. The last of several added convolutional layers to base feature extractor, in experiments [11] with COCO dataset [43], predicts three boxes at each scale, so the total YOLOv3′s output tensor for each different detector is N × N × [3 × (4 + 1 + 80)] for the 4 bounding box offsets, 1 objectness prediction, and 80 class predictions.

In the next generation of the YOLO model—YOLOv4 [12], as the optimal backbone model is determined CSPDarknet53 [48] (CSP stands for Cross Stage Partial). Modification is made in such a way that the last convolutional block in the Darknet-53 backbone network is improved to be a dense block [49]. The neck part of the YOLOv4 model consists of SPP (Spatial Pyramid Pooling) block [50,51] and a feature aggregation block. SPP block increases the receptive field and extracts the most important features of the CSPDarknet53 backbone output feature map. Feature aggregation block consists of modified Path Aggregation Network (PANet) [52] which is an advanced version of Feature Pyramid Network (FPN) [53] which is used in YOLOv3. In modified PANet architecture for YOLOv4, element-wise addition operation in shortcut connection is replaced with the concatenation operation. For the head in YOLOv4 is used the YOLOv3 anchor-based head and three levels of detection granularity.

Architecture of the next YOLO generation—YOLOv5 is very similar to the architecture of the YOLOv4. Although there is no published paper for YOLOv5, but only a repository on GitHub [13], the YOLOv5 model has been used in many studies [54,55]. Unlike previous versions of YOLO that are written in Darknet framework in the C programming language, YOLOv5 is written in Python which makes installation and integration much easier. Generally, YOLOv5 uses the CSPDarknet53 as the backbone, SPP block, and PANet as neck, and YOLOv3 detection head [11]. In order to avoid the procedure for selecting anchor box size and shape, using the k-mean clustering algorithm on the unique dataset as applied in previous versions and manual configuration in YOLO architecture, YOLOv5 introduces the function of adaptive anchor box calculation. The size of the optimal anchor box in different training sets is calculated adaptively during the training procedure.

YOLOv4 and YOLOv5 are anchor-based detectors with hand-crafted assigning rules for training, while new research in the object detection area is heading in the direction of anchor-free detectors [56,57,58]. Anchor-free detectors, advanced label assignment strategies, and end-to-end detectors triggered the development of the new YOLO model—YOLOX [14].

### YOLOX

As a starting point for the development of the YOLOX model, YOLOv3 is used.

YOLO models through the series evolved in different parts, but the head remained the same—coupled head. Studies in [14] have shown that the use of coupled heads harms performance and due to the conflict between classification and regression in the object detection task, the head in YOLOX is replaced with a decoupled head—Figure A1. Decoupled head separates the classification and localization operations. As shown in Figure A1, for each level of FPN feature, the decoupled head contains a 1 × 1 convolutional layer for channel dimension reduction followed by two 3 × 3 convolutional layers in two parallel branches. In both branches are then added per one 1 × 1 convolutional layer. Moreover, on the regression branch is added the IOU branch. This decoupled head improves the converging speed [14].

Unlike previous YOLO models, YOLOX switches to an anchor-free mechanism. In a very simple way, in YOLOX the number of predictions is reduced for each location from 3 to 1, thus it directly predicts two offsets of the bounding box top-left corner and the width and height.

In order to be consistent with the baseline YOLOv3, YOLOX selects one positive sample for each object—the central location of the object, and neglects other high-rate predictions. With an anchor-free mechanism, the detector’s training and decoding phase become simpler and the number of design parameters is reduced.

Selecting a single positive sample for each object, YOLOX ignores other high-quality predictions, while optimizing these high-quality predictions can also yield useful gradients, which can alleviate the extreme imbalance of positive/negative sampling during training [14]. The center 3 × 3 area is assigned as positives.

A necessary procedure for detector training is defining classification and regression targets for each anchor, which is named label assignment. Advanced label assignment in YOLOX is based on simplified OTA [56] which meets four key points in advanced label assignment: loss/quality-aware, center prior, dynamic number of positive anchors for each ground-truth (abbreviated as dynamic top-k) and global view.

In OTA [59], label assignment is treated as an Optimal Transport (OT) problem, analyzing the label assignment from a global perspective. Optimal Transport problem is solved with the Sinkhorn–Knopp algorithm [59], which requires 25% extra training time. Therefore, the Sinkhorn–Knopp algorithm is simplified to a dynamic top-k strategy, and the obtained approximate solution is called SimOTA [14]. In addition to reducing the detector training time, with SimOTA additional solver hyperparameters in the Sinkhorn–Knopp algorithm is avoided [14].

In order to obtain end-to-end YOLO, two additional convolutional layers are added. The first one is one-to-one label assignment and the second one is stop the gradient. However, these layers are left as an option due to a slight reduction in performance and speed of inference.
